# Wet Environmental Surveillance of Antimicrobial Resistance Genes in High-Risk Hospital Units

**DOI:** 10.3390/antibiotics15080807

**Published:** 2026-08-18

**Authors:** Morana Magaš, Bojana Mohar Vitezić, Kata Ivanišević, Maja Abram

**Affiliations:** 1 Department of Nursing, Faculty of Health Studies, University of Rijeka, Viktora Cara Emina 5, 51 000 Rijeka, Croatia; kata.ivanisevic@uniri.hr; 2 Department of Infection Prevention and Control, Clinical Hospital Center Rijeka, Krešimirova 42, 51 000 Rijeka, Croatia; 3 Department of Microbiology and Parasitology, Faculty of Medicine, University of Rijeka, Braće Branchetta 20, 51 000 Rijeka, Croatia; bojana.mohar@uniri.hr; 4 Department of Clinical Microbiology, Clinical Hospital Center Rijeka, Vjekoslava Dukića 7, 51 000 Rijeka, Croatia

**Keywords:** antimicrobial resistance genes, carbapenemase, ESBL, haematology, hospital wastewater, infection prevention and control, intensive care unit, point-prevalence environmental assessment, sink drains

## Abstract

**Background/Objectives**: Hospital water and wastewater environments are potential reservoirs for antimicrobial resistance genes (ARGs), particularly in high-risk clinical units. This study examined site-specific ARG DNA detection patterns in wet and waste-associated sites of a haematology ward and an intensive care unit (ICU) to identify environmental hotspots that may inform targeted infection prevention and control (IPC) interventions. **Methods**: A descriptive point-prevalence environmental study was conducted in May 2025 in a tertiary acute-care hospital. Samples were collected after routine cleaning and during usual clinical activity. Real-time PCR assays were used to detect *bla_TEM_*, *bla_SHV_*, *bla_CTX-M_*, *bla_OXA-48-like_*, *bla_VIM_*, *bla_NDM_*, *bla_IMP_*, *bla_KPC_*, and *mcr-1*. Analytical summaries included samples meeting predefined quality-control criteria. Ct signals were categorised descriptively; Ct values ≤ 40 were considered positive, with Ct values 36–40 classified as very low molecular signals, whereas Ct values > 40 were considered negative. **Results**: The analytical dataset comprised 60 samples: 31 from haematology and 29 from ICU. At least one included ARG signal was detected in 40/60 samples (66.7%), including 23/31 haematology samples (74.2%) and 17/29 ICU samples (58.6%), *bla_TEM_* (51.7%) and *bla_SHV_* (43.3%) were most frequent. Sink drains showed the broadest multi-gene profiles, especially combinations of ESBL- and carbapenemase-associated genes. Haematology showed broader carbapenemase-associated diversity, whereas ICU detections clustered in patient-room sink drains and sluice-room wastewater infrastructure. **Conclusions**: ARG DNA signals were unevenly distributed, with sink drains representing most frequently positive sampling sites for multi-gene detection. This molecular, single-timepoint environmental study does not demonstrate viable organisms, colonisation, or transmission; however, point-prevalence environmental assessments may identify wet and wastewater-associated hotspots that can inform targeted IPC interventions against the spread of multidrug-resistant microorganisms.

## 1. Introduction

Antimicrobial resistance (AMR) represents a significant global threat to patient safety, particularly in hospital settings with intensive antimicrobial consumption and high selective pressure [1,2]. Of special concern are multidrug-resistant Gram-negative bacteria (MDR-GNB) posing one of the greatest challenges to healthcare today because of their propensity for human-to-human transmission and lack of therapeutic options [3]. Traditionally, infection prevention and control (IPC) strategies have focused on hand hygiene, surface disinfection, and patient isolation [4]. Although these measures are essential, they primarily address direct contact transmission, while less emphasis has been placed on environmental niches, such as wet environments, drainage systems, and waste-fluid handling areas, that may support the long-term persistence and dissemination of antimicrobial resistance genes (ARGs) by horizontal gene transfer within microbial community.

Recent studies have highlighted the importance of hospital water systems and washbasin drainage infrastructure as reservoirs of MDR organisms, including carbapenemase-producing bacteria [5,6]. Nonetheless, most available studies focus on outbreak investigations or MDR clinical isolate identification and surveillance, while environmental data on ARGs in routine non-sterile hospital settings remain limited. This gap is particularly evident in comparative analyses of different high-risk clinical settings, where environmental antimicrobial pressure and patient care dynamics may vary substantially. The intensive care unit (ICU) and haematology wards are high-risk environments for MDR bacteria [7,8]. ICU patients undergo numerous invasive diagnostic and therapeutic procedures, frequently requiring central lines, mechanical ventilators, and urinary catheters, creating direct pathways for bacteria to enter the body. The haematology ward represents a particularly vulnerable hospital ecosystem. Severe immunosuppressed patients often experience prolonged hospital stays, prophylactic, empirical and specific combination antimicrobial therapies, creating conditions conducive to the selection and persistence of MDR microorganisms and their ARGs that are constantly spreading through horizontal gene transfer among the hospital microbial population [9,10]. In such settings, protective isolation measures are commonly implemented to protect patients from exogenous pathogens. However, while patient rooms may be subject to intensified cleaning and restricted access, associated sanitary infrastructure and auxiliary environmental systems may be overlooked, despite their potential role as resistance reservoirs. Of particular concern about the presence of AMR is the prevalence and dissemination of ARGs in wet hospital environments.

From the IPC perspective, understanding the spatial distribution of ARGs within the hospital environment is essential for environmental risk assessment and targeted prevention strategies. Identifying environmental hotspots associated with water systems and waste-fluid disposal supports risk-adapted interventions that complement existing patient-centred measures and contributes to a more comprehensive AMR control framework [11,12,13,14]. Understanding how ARGs are distributed across environmental sites may therefore provide insight into environmental risk patterns that remain insufficiently characterised in routine hospital settings.

Therefore, this study aimed to determine the presence and distribution of the most common extended-spectrum β-lactamase (ESBL)-encoding genes (*bla_TEM_*, *bla_SHV_*, and *bla_CTX-M_*), the most critical clinically relevant carbapenemase genes (*bla_OXA-48-like_*, *bla_VIM_*, *bla_NDM_*, *bla_IMP_*, and *bla_KPC_*), as well as mobilised colistin resistance (*mcr-1*) genes, in two high-risk hospital units, the haematology ward and ICU.

## 2. Results

### 2.1. Analytical Dataset

A total of 60 samples were collected from wet and waste-associated hospital indoor environments in two high-risk departments: 31 from haematology, and 29 from ICU (Table 1). Swab samples were collected from faucets (aerators and terminal rims), sink drains, toilet bowls (the interior sidewalls), and sluice-room waste-handling infrastructure (slop hopper).

Samples were analysed for the presence of resistance genetic markers using a culture-independent approach, detecting *bla*, and *mcr-1* genes via PCR. Genes encoding ESBLs (TEM, SHV, CTX-M), clinically important carbapenemases (OXA-48-like, VIM, NDM, IMP, KPC), and phosphoethanolamine transferase responsible for resistance to colistin and related polymyxins were assessed. As shown in Table 2, environmental samples from the indoor wet hospital environment are dominated by *bla* genes that code for the production of ESBLs (*bla_TEM_* 51.7%; *bla_SHV_* 43.3%; *bla_CTX-M_* 15%). Overall ARG abundance was higher in the haematology ward than in ICU, except for *bla_CTX-M_*, which is present in a higher percentage in ICU samples.

Regarding the presence of ARGs responsible for the production of carbapenemases, the percentage of positive samples is lower with the dominance of *bla_OXA-48-like_* and *bla_VIM_*. Although there is a relatively small number of positive samples, again, there is a higher prevalence of these genes in samples collected in the haematology department than in the ICU (Table 2). Also, *bla_KPC_* was detected in five screening samples from sink drains in haematology ward. According to the Ct values, a high copy number of the *bla_KPC_* gene was found in two (Ct 15–25), and moderate (Ct 26–30) in one environmental sample. In only one of the 60 analysed samples, the presence of the *mcr-1* gene responsible for resistance to colistin was proven, and that was in the haematology department, too. However, high cycle threshold (Ct = 38) value indicates a very low concentration of target ARGs in the sampled environment, demonstrating a low baseline abundance or trace environmental contamination of this antimicrobial resistance marker (Table 2). Supplementary Table S1 contains the cycle threshold (Ct) values for all environmental samples included in the study.

### 2.2. Site-Specific Environmental Patterns

Following the distribution of selected ARGs, depending on the sampling location, the most frequently positive site among wet environmental hotspots for ARGs related to sink drains with 76,2% positive samples (Table 3). As expected, ARGs show a higher abundance in sink drains compared to matched faucets, but, surprisingly, also in comparison to toilet bowls as well as slop hoppers (Table 3).

In addition to the higher prevalence, the sink drains are also characterised by a greater diversity of ARGs, as is clearly visible from Table 4. Also, the only sample in which the *mcr-1* gene was detected was obtained from one sink drain, confirming their role as dense ARG reservoirs.

## 3. Discussion

Healthcare-associated infections, due to antibiotic-resistant microorganisms, pose an acute and rising threat to critically ill and immunocompromised patients [1,2,8,9,10]. Resistant bacteria of special concern include critical priority pathogens like ESBL-producing and carbapenem-resistant Gram-negative bacilli, which are central targets of contemporary IPC efforts [3,8,10]. These microorganisms thrive in healthcare settings due to intense antimicrobial use and their ability to survive in different environmental niches [3,5,7,11,12,15,16,17]. Besides viable MDR-GNB, hospital indoor environments harbour a high abundance and diversity of ARGs that may persist even after routine cleaning and pose significant transmission risks for healthcare-associated infections [5,11,12,15,16,17]. Wet hospital environments, such as sink drains, U-bends, and plumbing systems, can act as reservoirs for microorganisms and ARGs because of biofilm formation, persistent moisture, nutrient accumulation, and opportunities for horizontal gene transfer [6,7,11,12,13,14,15,16,17].

Here, we performed PCR screening for ESBL and carbapenemase-associated ARGs, as well as *mcr-1*, by swabbing wet areas: faucets, sink drains, toilet bowls, and sluice-room waste-handling infrastructure, including the slop hopper, in two high-risk wards, haematology and ICU, of a tertiary university hospital.

The study highlights hospital sink drains as major reservoirs for ARGs, containing the highest abundance and diversity of clinically relevant ARGs compared with other screened wet environments [11,12,13,14,15,16,17]. Sinks may act as confluence points for nutrients, discarded body fluids, and antimicrobial residues [5,6,11,12]. The somewhat higher presence of ARGs in sink drains in comparison to matched faucets can be explained by constant nutrient accumulation and moisture in drains, whereas faucets have intermittent flow and exposure to treated water [6,11,12]. However, the higher contamination of sink drains compared with the inside of toilet bowls and the slop hopper specialised for patient waste disposal is somewhat unexpected but important from the IPC perspective [6,13,14].

Based on routine clinical practice in the participating units, most ICU patients were non-ambulatory and therefore had limited direct interaction with sinks and other sanitary facilities, whereas haematology patients who were clinically able routinely used shared or dedicated sanitary facilities for personal hygiene. This information provides clinical context for interpreting the environmental findings and should not be regarded as an observation formally evaluated in the present study.

Differences in the organisation and utilisation of sanitary facilities between the two units should also be considered when interpreting the observed environmental patterns. On the haematology ward, each isolation room had a dedicated patient sanitary facility, whereas most ICU patients were non-ambulatory and therefore had substantially less direct contact with sanitary facilities. Consequently, the observed environmental patterns may have been influenced not only by patient-related clinical factors but also by differences in the organisation and utilisation of sanitary facilities between the two units.

Among the targeted *bla* genes, ESBL-associated genes predominated, specifically *bla_TEM_* and *bla_SHV_* over *bla_CTX-M_*. Although *bla_SHV_* and *bla_TEM_* signals may represent ESBL or non-ESBL variants depending on the allele, the observed pattern of low CTX-M with higher SHV/TEM levels is more typical of older, narrow-spectrum penicillinases rather than CTX-M-dominant ESBLs. Without allele-level resolution, definitive classification is not possible [10]. Amplicon confirmation by sequencing, melting curve analysis, or gel electrophoresis was not performed in the present study. Consequently, allele-level discrimination of *bla_TEM_* and *bla_SHV_* signals was not possible, and the identity of amplified products was not independently confirmed. In addition, very low molecular signals (Ct 36–40) should be interpreted cautiously because they were not verified using independent confirmatory methods. Future studies combining environmental molecular monitoring with confirmatory sequencing would improve the characterisation of detected antimicrobial resistance determinants.

As for the “big five” carbapenemases, *bla_OXA-48-like_* and *bla_VIM_* were detected in nine out of 60 samples, *bla_NDM_* in six, and *bla_IMP_* and *bla_KPC_* in five samples each [3,8,17]. While bla_ESBL_ genes were uniformly distributed in both the haematology department and the ICU, it is concerning that bla_carbapenemase_ genes were mainly detected in samples collected in the haematology department. Immediate action and implementation of additional IPC measures were carried out after the simultaneous detection of strong signals for several *bla* genes, including *bla_CTX-M_*, *bla_TEM_*, *bla_SHV_*, *bla_KPC_*, and *bla_VIM_*, in the sink drain of one isolation room for haematological patients. Although the sink drain represented a potential environmental reservoir, no colonisation or infection with multidrug-resistant bacteria was identified in the patient occupying that room at the time of sampling or in patients admitted to the same room during the following six months after environmental screening. However, this observation was not part of the predefined study design and should not be interpreted as evidence against environmental transmission.

The *mcr-1* gene, which confers resistance to colistin [18], was detected in a single sink drain sample from the haematology ward. The very high Ct value (Ct 38) indicates a low target concentration consistent with trace environmental DNA [19,20]. As culture confirmation and sequencing were not performed, this isolated signal should be interpreted with caution and does not indicate the environmental dissemination of colistin resistance. Its detection is therefore considered a limited molecular finding that may warrant further investigation only if observed more consistently in future studies. Routine reviews of sink drain cleaning and disinfection practices remains important for preventing the accumulation of antimicrobial resistance determinants in wet environmental reservoirs [6,13,14].

Overall, ARG abundance and diversity were higher in the haematology ward than in the ICU. This may reflect the clinical context of haematology care, including prolonged hospital stays, long-term antibacterial prophylaxis regimens, repeated combined antimicrobial therapy, immunosuppression, recurrent intensive chemotherapy cycles, and repeated contacts with healthcare systems [9,10]. A hospital ICU also treats critically ill patients who need constant monitoring, advanced life support, and specialised medical care [8,13]. However, ICU patients rarely interact with sinks directly, whereas haematology patients frequently use room sinks for personal hygiene. This direct contact, combined with severe, prolonged immunosuppression, significantly increases their susceptibility to opportunistic, drain-borne infections [9,10,11,12,13,15,16]. The greater diversity of carbapenemase-associated genes observed in the haematology ward may reflect differences in patient characteristics and clinical care, including prolonged hospitalisation, repeated antimicrobial exposure, immunosuppression, and more frequent use of sanitary facilities than in ICU patients. Although these factors were not directly assessed in this study, they provide a plausible contextual framework for interpreting the observed environmental patterns.

An additional methodological limitation is the absence of normalisation to a universal bacterial-load target such as the 16S rRNA gene. Consequently, Ct values may reflect differences in both ARG abundance and total bacterial biomass. Therefore, differences observed between environmental sites, particularly those with substantially different biofilm burden such as sink drains and faucets, should be interpreted cautiously. Current environmental qPCR recommendations advocate normalisation of ARG abundance to 16S rRNA gene copies to distinguish true ARG enrichment from differences in overall bacterial load and improve comparability between sampling sites [21].

### 3.1. Translating Point-Prevalence Environmental Assessment into IPC Interventions

The potential translational value of environmental ARG screening is supported by clinical evidence showing that surveillance-driven infection-control bundles can reduce healthcare-associated infections caused by multidrug-resistant Gram-negative bacteria. A recent study demonstrated that weekly active surveillance, prompt culture notification, and standardised isolation audits reduced the incidence of hospital-acquired imipenem-resistant *Pseudomonas aeruginosa* from 1.842 to 0.963 cases per 1000 patient-days [22]. Furthermore, the persistence of carbapenemase-associated signals in haematology sink drains is consistent with the recognised role of hospital drainage systems as microbial reservoirs. Previous studies have shown that biofilm-forming imipenem-resistant *Pseudomonas aeruginosa* may persist within sink drains through quorum-sensing mechanisms that promote biofilm formation and surface adhesion [23]. Although the present study did not investigate *Pseudomonas aeruginosa* specifically, these findings support the rationale for drainage-focused IPC strategies and provide biological context for the persistence of antimicrobial resistance determinants in wet hospital environments.

Although it is still not a routine identification tool, point-prevalence environmental assessment may complement conventional IPC programmes by identifying wet and waste-associated locations that warrant increased attention despite appearing visually clean [2,5,11,12,15,16,17]. In the present study, ARG detection does not demonstrate transmission but highlights environmental sites where patient care, wastewater exposure, and sanitary infrastructure intersect [19,20]. These findings support targeted environmental risk assessment, staff education, reinforcement of safe sink use, safe disposal of patient care fluids, separation of clean supplies from sink splash zones, and environmental hygiene practices [3,4,6,13,14,24].

Effective mitigation requires collaboration between IPC professionals, nursing and clinical staff, microbiology laboratories, environmental services, and facility management, particularly when considering interventions directed at drainage systems and wastewater infrastructure [3,4,6,14,24]. Any drain-focused intervention should be implemented according to local policy, product instructions, occupational safety requirements, and follow-up monitoring [6,14].

### 3.2. Limitations

Our study has several limitations. Environmental sampling was performed during a single sampling period, and each predefined environmental sampling site was sampled only once. Although all samples were collected using an identical protocol by the same investigator, some variability in the amount of collected material cannot be excluded. PCR detected ARG DNA and did not confirm viable bacteria, bacterial species, alleles, plasmids, patient colonisation, or transmission. The absence of sequencing precluded the allele-level discrimination of *bla_TEM_* and *bla_SHV_* amplification products. Consequently, these targets were interpreted at the gene-family level without assigning specific ESBL or non-ESBL variants.

Environmental sampling was performed during a single sampling period; therefore, temporal variation in environmental contamination throughout the day could not be assessed. Furthermore, because the molecular assays detected environmental DNA rather than viable microorganisms, the findings cannot distinguish between DNA originating from viable or non-viable bacteria, nor can they be interpreted as evidence of bacterial transmission or healthcare-associated infection.

Furthermore, patient colonisation and infection data were not collected as part of the predefined study protocol. Therefore, no direct association between environmental ARG detection and contemporaneous clinical isolates could be established.

Repeated sampling, culture, sequencing, and comparison with clinical isolates would be needed to evaluate persistence and transmission more directly.

## 4. Materials and Methods

### 4.1. Study Design, Setting, and Sampling

This descriptive point-prevalence environmental study was conducted in May 2025 in a tertiary acute-care hospital. The haematology ward comprised 18 beds, including six single-bed isolation-room beds and 12 beds in multi-bed patient rooms. Each isolation room had a dedicated sanitary facility equipped with a toilet and handwashing sink, whereas patients accommodated in multi-bed rooms shared separate male and female sanitary facilities. Environmental sampling on the haematology ward included sink drains, sink faucets, and toilet bowls from both dedicated isolation-room and shared patient sanitary facilities. The ICU comprised 14 beds, including three four-bed patient rooms and one two-bed patient room. Environmental sampling included patient rooms, patient sanitary facilities, staff sanitary facilities, and the separate sluice room containing the slop hoppers. Because ICU patients were predominantly non-ambulatory, sinks located within patient care areas were used primarily by healthcare staff rather than by patients. At the time of sampling, all rooms and beds were occupied except ICU patient room 3. Because environmental sampling sites were selected purposively and the study was designed as a descriptive point-prevalence survey, no inferential statistical comparisons between environmental locations were planned.

Environmental samples were collected at a predefined time point within the routine clinical workflow, following routine morning patient care and environmental cleaning under normal operating conditions. Routine cleaning and disinfection of rooms and sanitary facilities was performed at approximately 8:30. Environmental sampling was performed afterwards, at approximately 10:00, after sufficient time had elapsed following routine cleaning to minimise the influence of the immediate effect of cleaning and disinfection procedures. This timing represents a post-cleaning, early-use phase of the hospital environment under routine operating conditions, but should not be interpreted as a stable or universal daily baseline of environmental contamination.

A total of 60 environmental samples were collected from sink drains, faucets, toilet bowls, and ICU sluice-room waste-handling infrastructure.

A predefined sampling procedure was developed before environmental sampling commenced and applied consistently throughout the study. Environmental sampling was performed using sterile FLOQ swabs (Copan, Brescia, Italy). Two swabs were collected from each site and placed into one tube containing 1 mL sterile saline solution. For drain-associated sites, the swab was inserted approximately 5 cm into the upper accessible portion of the drain and moved vertically along the inner surfaces using consistent up-and-down scrubbing strokes. Faucet aerators were sampled by wiping the whole external surface without disassembly. Toilet bowls and sluice hoppers were sampled after flushing by wiping the inner surface above the waterline while avoiding direct contact with water. All samples from individual predetermined environmental locations were collected by rotating the swab to ensure full contact with the sampled surface, following an identical sampling protocol performed by the same trained investigator. Samples were transported immediately to the microbiology laboratory and processed within two hours of collection.

### 4.2. DNA Extraction

DNA extraction from environmental samples was performed using the SaMag Bacterial DNA Extraction Kit (SM006) on the SaMag-12 automated extraction system (Sacace Biotechnologies, Como, Italy), using a modified protocol adapted for environmental samples. Prior to extraction, samples were sonicated in an ultrasonic bath for 2 min at 40 kHz. After sonication, the entire liquid volume was centrifuged at 13,000 rpm for 10 min. The supernatant was discarded, and approximately 100 µL of sediment was resuspended in 300 µL lysis buffer (BLB2) and vortexed. Samples were then incubated on a thermomixer (Eppendorf, Hamburg, Germany) for 1 h at 70 °C with shaking at 1000 rpm. After incubation, 400 µL of the processed sample was transferred into sample tubes on the SaMag sample rack, and nucleic acid isolation proceeded according to the manufacturer’s protocol (input volume 400 µL; elution volume 100 µL).

Extracted DNA concentrations were measured using the Qubit™ Fluorometer with the Qubit^®^ dsDNA HS Assay Kit (Invitrogen™, Thermo Fisher Scientific, Waltham, MA, USA) according to the manufacturer’s instructions.

### 4.3. Molecular Analysis

Molecular detection of antimicrobial resistance genes was performed using real-time PCR assays. Detection of ESBL-associated genes (*bla_CTX-M_*, *bla_TEM_*, *bla_SHV_*) and the colistin resistance gene (*mcr-1*) was carried out using the VIASURE CTX, TEM, SHV and mcr Real-Time PCR Detection Kit (CerTest Biotec, Zaragoza, Spain). Detection of carbapenemase genes (*bla_OXA-48-like_*, *bla_KPC_*, *bla_IMP_*, *bla_NDM_*, *bla_VIM_*) was performed using the VIASURE Carbapenemase-Producing Enterobacteriaceae Real-Time PCR Detection Kit (CerTest Biotec, Zaragoza, Spain), according to the manufacturer’s protocol, using a QuantStudio Real-Time PCR System (Thermo Fisher Scientific, Waltham, MA, USA).

The analytical sensitivity reported by the manufacturer for the *bla_CTX_*, *bla_TEM_*, *bla_SHV_* and *mcr-1* Real-Time PCR Detection Kit was 0.02 CFU per reaction for CTX (*bla_CTX-M1_*, *bla_CTX_*_-M9_ [Cluster A or *bla_CTX-M-A_*] and *bla_CTX-M2_*, *bla_CTX-M8_*, *bla_CTX-M-25_* [Cluster B or *bla_CTX-M-B_*]), 0.08 CFU per reaction for TEM (*bla_TEM_*), 8.75 DNA copies per reaction for SHV (*bla_SHV_*), and 0.02 CFU per reaction for *mcr 1*, with a reported positive detection rate of 95%. The Carbapenemase-Producing Enterobacteriaceae Real-Time PCR Detection Kit had a detection limit of 10 DNA copies per reaction for NDM, KPC and IMP, 50 DNA copies per reaction for *bla_OXA-48-like_*, and 100 DNA copies per reaction for *bla_VIM_*.

Ct values ≤ 40 were considered positive and were included in the descriptive analyses, whereas Ct values > 40 were considered negative. Ct values between 36 and 40 were predefined as very low molecular signals and evaluated according to the Ct categories established prior to analysis. These very low molecular signals were interpreted conservatively and were not used in analytical steps implying viable organisms, transmission events, or environmental dissemination.

PCR analyses were performed according to the laboratory’s routine protocol and the manufacturer’s instructions for the CE-IVD assays. Positive reactions were interpreted according to the manufacturer’s assay criteria. Additional replicate or repeat testing was not incorporated into the predefined laboratory protocol.

All PCR reactions included an internal amplification control to monitor PCR inhibition and ensure reliable amplification. Negative controls without template DNA and positive control reactions provided within the assay kits were included in each run according to the manufacturer’s protocol to verify assay performance and exclude contamination.

Amplification results were recorded as cycle threshold (Ct) values for each target gene. Lower Ct values indicate earlier detection and stronger amplification signal, whereas higher Ct values indicate later detection and lower abundance. According to the predefined Ct categories, amplification signals were classified into four groups based on predefined intervals: strong amplification signal (High, Ct 15–25), moderate amplification signal (Moderate, Ct 26–30), low amplification signal (Low, Ct 31–35), and very low signal (Very Low, Ct 36–40). A ‘not detected’ (ND) report corresponds to Ct = 0, and Ct > 40 and is equivalent to a negative result.

Because sequencing was not performed, *bla_TEM_* and *bla_SHV_* signals could not be assigned to specific ESBL or non-ESBL alleles. Therefore, these findings were interpreted at the β-lactamase gene-family level without assigning specific ESBL or non-ESBL variants.

### 4.4. AI Statement

Generative artificial intelligence tools were used exclusively for language editing and formatting improvement. No AI-generated data, analyses, or interpretations were included in this study.

## 5. Conclusions

ARG DNA signals were unevenly distributed across wet and waste-associated environments in the haematology ward and ICU. Sink drains showed the clearest multi-gene detection patterns, including combinations of ESBL-associated and carbapenemase-associated genes, whereas the haematology ward demonstrated greater carbapenemase-associated gene diversity than the ICU.

These findings should be interpreted considering the study’s limitations, including single-time-point environmental sampling, detection of environmental DNA rather than viable microorganisms, and the absence of evidence linking environmental findings to bacterial transmission or healthcare-associated infections.

Nevertheless, targeted environmental assessment of sink drains may complement local IPC programmes by supporting environmental risk assessment and identifying locations that warrant further investigation in high-risk hospital units. Future longitudinal environmental molecular monitoring studies are needed to determine how these findings can best inform targeted environmental interventions and routine environmental management practices.

## Figures and Tables

**Table 1:** Sampling framework and analytical dataset.

Unit	Setting	Sampled Environments	Collected Samples
Haematology	18 beds: 6 isolation-room beds, 12 ward beds	Faucets, sink drains, toilet bowls from isolation-room and shared patient sanitary facilities	31
ICU	14 beds: four patient rooms; patient sanitary facilities, staff sanitary facilities, sluice room	Faucets, sink drains, toilet bowls, and sluice-room waste-handling infrastructure	29
Overall		Wet and waste-associated environmental sites	60

**Table 2:** Gene-specific detection frequency.

Gene	Overall	Haematology	ICU	Ct Value Ranges	Signal Categories (H; M; L; VL) *
*bla_TEM_*	31/60 (51.7%)	18/31 (58.1%)	13/29 (44.8%)	20–40	H 3; M 4; L 9; VL 15; ND 29
*bla_SHV_*	26/60 (43.3%)	16/31 (51.6%)	10/29 (34.5%)	21–40	H 3; M 4; L 9; VL 10; ND 34
*bla_CTX-M_*	9/60 (15.0%)	3/31 (9.7%)	6/29 (20.7%)	20–40	H 4; M 2; L 1; VL 2; ND 51
*bla_OXA-48-like_*	9/60 (15.0%)	5/31 (16.1%)	4/29 (13.8%)	17–38	H 2; M 1; L 3; VL 3; ND 51
*bla_VIM_*	9/60 (15.0%)	6/31 (19.4%)	3/29 (10.3%)	17–35	H 6; M 2; L 1; ND 51
*bla_NDM_*	6/60 (10.0%)	5/31 (16.1%)	1/29 (3.4%)	27–35	M 2; L 4; ND 54
*bla_IMP_*	5/60 (8.3%)	4/31 (12.9%)	1/29 (3.4%)	30–37	M 1; L 3; VL 1; ND 55
*bla_KPC_*	5/60 (8.3%)	5/31 (16.1%)	0/29 (0.0%)	18–35	H 2; M 1; L 2; ND 55
*mcr-1*	1/60 (1.7%)	1/31 (3.2%)	0/29 (0.0%)	38	VL 1; ND 59

* Legend. H: Ct 15–25, strong positive, high target concentration; M: Ct 26–30, moderate positive, moderate target concentration; L: Ct 31–35, low positive, low target concertation; VL: Ct 36–40, very low signal, very low target concentration; ND: Ct 0 and Ct > 40, target not detected.

**Table 3:** Site-specific ARG detection patterns.

Site Category	No. Samples	ARGs Positivity	Ct Categories (H; M; L; VL; ND) *	Specific ARGs (No. Positive Samples)
Sink drain	21	16/21 (76.2%)	H 15; M 14; L 17; VL 8; ND 135	*bla_SHV_* (14), *bla_TEM_* (14), *bla_CTX-M_* (5), *bla_OXA-48-like_* (5), *bla_VIM_* (5), *bla_KPC_* (4)
Faucet	20	12/20 (60.0%)	H 0; M 0; L 3; VL 16; ND 161	*bla_TEM_* (10), *bla_SHV_* (4), *bla_CTX-M_* (2), *bla_IMP_* (1), *bla_NDM_* (1), *bla_OXA-48-like_* (1)
Toilet bowl	14	10/14 (71.4%)	H 1; M 2; L 12; VL 7; ND 104	*bla_TEM_* (7), *bla_SHV_* (6), *bla_VIM_* (3), *bla_OXA-48-like_* (2), *bla_CTX-M_* (1), *bla_IMP_* (1)
Sluice-room waste-handling infrastructure	5	2/5 (40.0%)	H 4; M 1; L 0; VL 1; ND 39	*bla_SHV_* (2), *bla_CTX-M_* (1), *bla_IMP_* (1), *bla_OXA-48-like_* (1), *bla_VIM_* (1)

* Legend. H: Ct 15–25, strong positive, high target concentration; M: Ct 26–30, moderate positive, moderate target concentration; L: Ct 31–35, low positive, low target concertation; VL: Ct 36–40, very low signal, very low target concentration; ND: Ct 0 and Ct > 40, target not detected.

**Table 4:** Number of included ARGs-positive signals across environmental sampling sites.

Sampling Site	*bla* *_TEM_*	*bla* *_SHV_*	*bla* *_CTX-M_*	*bla* *_OXA-48-like_*	*bla_VIM_*	*bla* *_NDM_*	*bla* *_IMP_*	*bla* *_KPC_*	*mcr-1*
Sink drain	14	14	5	5	5	4	2	4	1
Faucet	10	4	2	1	0	1	1	0	0
Toilet bowl	7	6	1	2	3	1	1	1	0
Sluice-room waste-handling infrastructure	0	2	1	1	1	0	1	0	0

Note: Cell shading represents a visual gradient (heat map) reflecting ARG abundance. The shading intensity corresponds directly to gene counts, ranging from the lightest shade for zero (0) values to the darkest shade for the highest counts, visually highlighting the sink drain as a dense ARG reservoir.

## Data Availability

The data supporting the findings of this study are available from the corresponding author upon reasonable request.

## Supplementary Materials

The following supporting information can be downloaded at: https://www.mdpi.com/article/10.3390/antibiotics15080807/s1, Table S1: Cycle threshold (Ct) values for all environmental samples included in the study.

## Abbreviations

The following abbreviations are used in this manuscript:

AMR	Antimicrobial resistance
ARG	Antimicrobial resistance gene
Ct	Cycle threshold
ESBL	Extended-spectrum beta-lactamase
ICU	Intensive care unit
IPC	Infection prevention and control
MDR-GNB	Multidrug-resistant Gram-negative bacteria
PCR	Polymerase chain reaction

## References

[1] Kariuki S. (2024). Global burden of antimicrobial resistance and forecasts to 2050. Lancet.

[2] Bertagnolio S., Dobreva Z., Centner C.M., Olaru I.D., Donà D., Burzo S., Huttner B.D., Chaillon A., Gebreselassie N., Wi T. (2024). WHO global research priorities for antimicrobial resistance in human health. Lancet Microbe.

[3] Mills J.P., Marchaim D. (2021). Multidrug-resistant Gram-negative bacteria: Infection prevention and control update. Infect. Dis. Clin. N. Am..

[4] Abbas S., Stevens M.P. (2024). Horizontal versus vertical strategies for infection prevention: Current practices and controversies. Curr. Opin. Infect. Dis..

[5] Silvester R., Perry W.B., Webster G., Rushton L., Baldwin A., Pass D.A., Byrnes N.A., Farkas K., Heginbothom M., Craine N. (2025). Metagenomic profiling of hospital wastewater: A comprehensive national scale analysis of antimicrobial resistance genes and opportunistic pathogens. J. Infect..

[6] Centers for Disease Control and Prevention Considerations for Reducing Risk: Water in Healthcare Facilities. Updated 6 February 2026. https://www.cdc.gov/healthcare-associated-infections/php/toolkit/water-management.html.

[7] Taner F., Baddal B., Theodoridis L., Petrovski S. (2024). Biofilm production in intensive care units: Challenges and implications. Pathogens.

[8] Dessemon J., Vacheron C.H., Savey A., Machut A., Friggeri A., Prevot C., Bourge X., Lepape A., Elias C., REA-REZO Study Group (2025). The impact of carbapenem-resistant infections in intensive care units: Focus on non-fermenting Gram-negative bacilli and survival analysis. Antimicrob. Resist. Infect. Control.

[9] Amanati A., Sajedianfard S., Khajeh S., Ghasempour S., Mehrangiz S., Nematolahi S., Shahhosein Z. (2021). Bloodstream infections in adult patients with malignancy, epidemiology, microbiology, and risk factors associated with mortality and multi-drug resistance. BMC Infect. Dis..

[10] Gallardo-Pizarro A., Lopera C., Peyrony O., Monzo-Gallo P., Aiello T.F., Martinez-Urrea A., Herrera S., Del Río A., Teijon-Lumbreras C., Chumbita M. (2025). Rectal colonization by multidrug-resistant Gram-negative bacteria and subsequent bacteraemia in haematological patients. Clin. Microbiol. Infect..

[11] Inkster T. (2024). A narrative review and update on drain-related outbreaks. J. Hosp. Infect..

[12] Laço J., Martorell S., Gallegos M.D.C., Gomila M. (2025). Yearlong analysis of bacterial diversity in hospital sink drains: Culturomics, antibiotic resistance and implications for infection control. Front. Microbiol..

[13] Kearney A., Boyle M.A., Curley G.F., Humphreys H. (2021). Preventing infections caused by carbapenemase-producing bacteria in the intensive care unit: Think about the sink. J. Crit. Care.

[14] Ali S., Burke L.P., Fitzpatrick F., Fitzgerald-Hughes D. (2026). A scoping review of disinfection strategies for carbapenemase-producing Enterobacterales in hospital water systems. J. Hosp. Infect..

[15] Constantinides B., Chau K.K., Quan T.P., Rodger G., Andersson M.I., Jeffery K., Lipworth S., Gweon H.S., Peniket A., Pike G. (2020). Genomic surveillance of *Escherichia coli* and *Klebsiella* spp. in hospital sink drains and patients. Microb. Genom..

[16] Diorio-Toth L., Wallace M.A., Farnsworth C.W., Wang B., Gul D., Kwon J.H., Andleeb S., Burnham C.D., Dantas G. (2023). Intensive care unit sinks are persistently colonized with multidrug resistant bacteria and mobilizable, resistance-conferring plasmids. mSystems.

[17] Park S.C., Parikh H., Vegesana K., Stoesser N., Barry K.E., Kotay S.M., Dudley S., Peto T.E.A., Crook D.W., Walker A.S. (2020). Risk factors associated with carbapenemase-producing Enterobacterales positivity in the hospital wastewater environment. Appl. Environ. Microbiol..

[18] Liu Y.Y., Wang Y., Walsh T.R., Yi L.X., Zhang R., Spencer J., Doi Y., Tian G., Dong B., Huang X. (2016). Emergence of plasmid-mediated colistin resistance mechanism MCR-1 in animals and human beings in China. Lancet Infect. Dis..

[19] Bustin S.A., Benes V., Garson J.A., Hellemans J., Huggett J., Kubista M., Mueller R., Nolan T., Pfaffl M.W., Shipley G.L. (2009). The MIQE guidelines: Minimum information for publication of quantitative real-time PCR experiments. Clin. Chem..

[20] Kralik P., Ricchi M. (2017). A basic guide to real-time PCR in microbial diagnostics: Definitions, parameters, and everything. Front. Microbiol..

[21] Keenum I., Liguori K., Calarco J., Davis B.C., Milligan E., Harwood V.J., Pruden A. (2022). A framework for standardized qPCR-targets and protocols for quantifying antibiotic resistance in surface water, recycled water and wastewater. Crit. Rev. Environ. Sci. Technol..

[22] Bhusal C.K., Rasheed H.J. (2026). Controlling Imipenem-Resistant *Pseudomonas aeruginosa* in Hospitals: Effect of Improved Infection Control Measures from an Interrupted Time-Series Study. World J. Exp. Biosci..

[23] Talib M.M., Attia M.S., Abdulrraziq A.A. (2026). Association of Imipenem Resistance with *LasR/RhlR* Quorum Sensing, Biofilm Formation, and Adhesion to Human Epithelial Cells in *Pseudomonas aeruginosa*. World J. Exp. Biosci..

[24] Mitchell B.G., Hall L., MacBeth D., Gardner A., Halton K. (2015). Hospital infection control units: Staffing, costs, and priorities. Am. J. Infect. Control.

