# Supplementary material for: Wet Environmental Surveillance of Antimicrobial Resistance Genes in High-Risk Hospital Units
Source: Antibiotics (Basel). 2026 Aug 18;15(8):807. doi: 10.3390/antibiotics15080807 (PMC13510240; doi:10.3390/antibiotics15080807)
Supplement: Supplementary file 1 [file antibiotics-15-00807-s001.zip › antibiotics-4497654-supplementary.pdf]

Supplementary Table S1. Cycle threshold (Ct) values for all environmental samples included in the study.

Supplementary Table S1. Cycle threshold (Ct) values for all environmental samples included in the study. Ct values >40 were considered negative, whereas Ct values between 36 and 40 were classified as very low molecular signals according to the predefined study categories described in the manuscript. DNA concentration measured after extraction is provided for reference only.

Sample ID	Ward	Sampling location	Surface	DNA concentration (ng/µL)	Ct blaCTX-M	Ct blaTEM	Ct blaSHV	Ct mcr-1	Ct blaOXA-48-like	Ct blaKPC	Ct blaIMP	Ct blaNDM	Ct blaVIM
1	HEM	Male patient sanitary facility	Toilet bowl	16.00	ND	35	34	ND	ND	ND	ND	ND	ND
2	HEM	Male patient sanitary facility	Sink drain	13.00	30	30	33	ND	ND	ND	ND	35	25
3	HEM	Male patient sanitary facility	Faucet	0.04	ND	ND	ND	ND	ND	ND	ND	ND	ND
4	HEM	Male patient sanitary facility	Toilet bowl	19,90	ND	ND	ND	ND	ND	ND	ND	ND	ND
5	HEM	Male patient sanitary facility	Sink drain	0,20	ND	30	30	ND	ND	ND	ND	ND	ND
6	HEM	Male patient sanitary facility	Sink drain	0,14	ND	ND	ND	ND	ND	ND	ND	ND	ND

7	HEM	Male patient sanitary facility	Faucet	0,80	ND	ND	ND	ND	ND	ND	ND	ND	ND
8	HEM	Male patient sanitary facility	Faucet	0,30	ND	ND	ND	ND	ND	ND	ND	ND	ND
9	HEM	Female patient sanitary facility	Toilet bowl	0,03	ND	ND	ND	ND	ND	ND	ND	ND	ND
10	HEM	Female patient sanitary facility	Toilet bowl	22,00	ND	35	34	ND	38	ND	ND	ND	23
11	HEM	Female patient sanitary facility	Sink drain	1,37	ND	31	32	ND	ND	35	ND	ND	ND
12	HEM	Female patient sanitary facility	Sink drain	8,08	ND	35	32	ND	ND	35	ND	ND	ND
13	HEM	Female patient sanitary facility	Faucet	0,50	ND	40	43	ND	ND	ND	ND	ND	ND
14	HEM	Female patient sanitary facility	Faucet	0,09	ND	38	ND	ND	ND	ND	ND	ND	ND

15	HEM	Patient isolation room 1	Toilet bowl	0,08	ND	ND	36	ND	ND	ND	ND	ND	ND
16	HEM	Patient isolation room 1	Sink drain	16,10	ND	ND	31	ND	ND	18	ND	ND	17
17	HEM	Patient isolation room 1	Faucet	0,60	ND	ND	ND	ND	ND	ND	ND	ND	ND
18	HEM	Patient isolation room 2	Toilet bowl	0,57	ND	41	41	ND	ND	30	ND	ND	27
19	HEM	Patient isolation room 2	Sink drain	1,51	20	22	21	ND	ND	25	ND	ND	25
20	HEM	Patient isolation room 2	Faucet	0,14	ND	ND	ND	ND	ND	ND	37	ND	ND
21	HEM	Patient isolation room 3	Toilet bowl	0,09	ND	ND	36	ND	ND	ND	ND	ND	35
22	HEM	Patient isolation room 3	Sink drain	too high	ND	29	27	38	29	ND	33	27	ND
23	HEM	Patient isolation room 3	Faucet	0,37	40	40	41	ND	ND	ND	ND	ND	ND
24	HEM	Patient isolation room 4	Toilet bowl	0,07	ND	36	41	ND	ND	ND	ND	ND	ND
25	HEM	Patient isolation room 4	Sink drain	0,03	ND	39	40	ND	ND	ND	ND	ND	ND

26	HEM	Patient isolation room 4	Faucet	0,07	ND	39	40	ND	ND	ND	ND	ND	ND
27	HEM	Patient isolation room 5	Toilet bowl	too high	ND	32	32	ND	35	ND	31	33	ND
28	HEM	Patient isolation room 5	Sink drain	0,56	ND	37	42	ND	ND	ND	ND	ND	ND
30	HEM	Patient isolation room 6	Toilet bowl	0,13	ND	ND	ND	ND	ND	ND	ND	ND	ND
31	HEM	Patient isolation room 6	Sink drain	too high	ND	33	33	ND	35	ND	35	31	ND
32	HEM	Patient isolation room	Faucet	0,03	ND	37	39	ND	38	ND	ND	35	ND
33	ICU	Patient isolation room	Sink drain	6,96	ND	ND	ND	ND	ND	ND	ND	ND	ND
34	ICU	Patient isolation room	Faucet	1,40	ND	38	ND	ND	ND	ND	ND	ND	ND
35	ICU	Patient room 3	Sink drain	0,30	ND	ND	ND	ND	ND	ND	ND	ND	ND
36	ICU	Patient room 3	Faucet	1,19	ND	ND	ND	ND	ND	ND	ND	ND	ND
37	ICU	Patient room 3	Sink drain	3,93	ND	ND	ND	ND	ND	ND	ND	ND	ND
38	ICU	Patient room 3	Faucet	9,91	ND	40	ND	ND	ND	ND	ND	ND	ND

39	ICU	Sluice room	Slop hopper drain	0,60	ND	ND	ND	ND	ND	ND	ND	ND	ND
40	ICU	Sluice room	Slop hopper faucet	1,05	ND	ND	ND	ND	ND	ND	ND	ND	ND
41	ICU	Sluice room	Slop hopper drain	4,92	21	ND	24	ND	17	ND	ND	ND	ND
42	ICU	Sluice room	Slop hopper faucet	0,20	ND	ND	ND	ND	ND	ND	ND	ND	ND
43	ICU	Sluice room	Slop hopper bowl	1,24	ND	ND	38	ND	ND	ND	30	ND	25
44	ICU	Patient room 2	Sink drain	0,40	ND	ND	ND	ND	ND	ND	ND	ND	ND
45	ICU	Patient room 2	Faucet	4,02	ND	ND	37	ND	ND	ND	ND	ND	ND
46	ICU	Patient room 2	Sink drain	35,20	20	20	26	ND	32	ND	ND	ND	19
47	ICU	Patient room 2	Faucet	0,08	37	38	ND	ND	ND	ND	ND	ND	ND
49	ICU	Staff sanitary facility	Faucet	0,30	ND	34	35	ND	ND	ND	ND	ND	ND
50	ICU	Staff sanitary facility	Toilet bowl	4,90	ND	35	42	ND	ND	ND	ND	ND	ND
51	ICU	Staff sanitary facility	Sink drain	22,00	ND	35	36	ND	ND	ND	ND	ND	ND

52	ICU	Staff sanitary facility	Faucet	0,60	ND	39	ND	ND	ND	ND	ND	ND	ND
53	ICU	Female patient sanitary facility	Sink drain	27,80	ND	ND	38	ND	ND	ND	ND	29	28
54	ICU	Female patient sanitary facility	Faucet	8,16	ND	ND	ND	ND	ND	ND	ND	ND	ND
55	ICU	Female patient sanitary facility	Toilet bowl	30,50	ND	38	38	ND	ND	ND	ND	ND	ND
56	ICU	Male patient sanitary facility	Sink drain	8,00	ND	38	ND	ND	ND	ND	ND	ND	ND
57	ICU	Male patient sanitary facility	Faucet	0,60	ND	ND	ND	ND	ND	ND	ND	ND	ND
58	ICU	Male patient sanitary facility	Toilet bowl	0,20	ND	ND	ND	ND	ND	ND	ND	ND	ND
59	ICU	Male patient sanitary facility	Toilet bowl	1,00	32	37	ND	ND	ND	ND	ND	ND	ND
60	ICU	Patient room 1	Sink drain	5,22	22	22	23	ND	18	ND	ND	ND	ND

62	ICU	Patient room 1	Sink drain	13,47	29	26	29	ND	37	ND	ND	ND	ND
63	ICU	Patient room 1	Faucet	0,80	ND	ND	ND	ND	ND	ND	ND	ND	ND

Abbreviations: HEM, haematology ward; ICU, intensive care unit; Ct, cycle threshold; ND, not detected.
